# L523S, an RNA-binding protein as a potential therapeutic target for lung cancer

**DOI:** 10.1038/sj.bjc.6600806

**Published:** 2003-03-18

**Authors:** T Wang, L Fan, Y Watanabe, P D McNeill, G G Moulton, C Bangur, G R Fanger, M Okada, Y Inoue, D H Persing, S G Reed

**Affiliations:** 1 Department of Tumor Antigen Discovery, Corixa Corporation, 1124 Columbia Street, Seattle, WA 98104, USA; 2 National Kinki-Chuo Hospital, 1180 Nagasone-Cho, Sakai City, Osaka, 591-8555, Japan; 3 Department of Pathobiology, University of Washington, Seattle, WA 98195, USA

**Keywords:** lung cancer, KOC RNA binding protein, therapeutic vaccine, tumor antigen

## Abstract

Approaches to vaccine-based immunotherapy of human cancer may ultimately require targets that are both tumour-specific and immunogenic. In order to generate specific antitumour immune responses to lung cancer, we have sought lung cancer-specific proteins that can be targeted for adjuvant vaccine therapy. By using a combination of cDNA subtraction and microarray analysis, we previously reported the identification of an RNA-binding protein within the KOC family, L523S, to be overexpressed in squamous cell cancers of the lung. We show here that L523S exhibits significant potential for vaccine immunotherapy of lung cancer. As an oncofetal protein, L523S is normally expressed in early embryonic tissues, yet it is re-expressed in a high percentage of nonsmall cell lung carcinoma. The specificity of L523S expression in lung cancer was demonstrated by both mRNA and protein measurements using real-time PCR, Western blot, and immunohistochemistry analyses. Furthermore, we show that immunological tolerance of L523S is naturally broken in lung cancer patients, as evidenced by detectable antibody responses to recombinant L523S protein in eight of 17 lung pleural effusions from lung cancer patients. Collectively, our studies suggest that L523S may be an important marker of malignant progression in human lung cancer, and further suggest that treatment approaches based on L523S as an immunogenic target are worthy of pursuit.

Among all human cancers, carcinoma of the lung has the highest mortality rate and is the leading cause of all cancer deaths. Although the majority of patients diagnosed with most histological types of lung cancer respond initially to conventional therapies, they often relapse within a relative short period of time, leaving no significant improvement in survival. Immunotherapy of lung cancer has been actively pursued in recent years (Bunn, 1999). However, randomised trials using nonspecific immune stimulants such as interferon have failed to show significant effects on lung cancer survival (Jett *et al*, 1994; van Zandwijk *et al*, 1997). Most pioneering studies of targeted cancer vaccine therapy have focused on melanoma, which is the most immunogenic solid tumour and has abundantly expressed melanoma-specific gene products. Clinical trials using antigens that are specifically expressed or overexpressed in melanomas are currently under investigation and these vaccines are targeted for humoral (B cell) and/or cellular responses (cytotoxic T cells) (Robbins and Kawakami, 1996; Brinckerhoff *et al*, 2000). The success of therapeutic cancer vaccines may ultimately rely on the identification of immunogenic antigens that are overexpressed in tumours relative to essential normal tissues.

In an effort to identify tumour antigens that can be targeted for specific immunotherapy, we used a high throughput method for identifying genes overexpressed in lung squamous cell carcinoma (LSCC) (Wang *et al*, 2000). Among 17 candidates found to be overexpressed in LSCC, we described one candidate, L523S, which has now risen to lead status. L523S was initially identified as a gene encoding a KOC RNA-binding protein that is overexpressed in pancreatic cancer (Mueller-Pillasch *et al*, 1997). Also known as IMP-3, L523S binds and regulates IGF-II transcripts during embryogenesis (Nielsen *et al*, 1999). In this report, we describe additional evaluation of L523S as a lung cancer vaccine candidate.

## MATERIALS AND METHODS

### Tissue, lung pleural effusion, and RNA sources

Tumour and normal tissues used in this study were obtained from Cooperative Human Tissue Network (CHTN), National Disease Research Interchange (NDRI), and Roswell Park Cancer Center. Other normal tissues were purchased from Clontech (Palo Alto, CA, USA) and Invitrogen (Carlsbad, CA, USA). Lung pleural effusions used in this study come from advanced stage IV patients with nonsmall cell lung carcinoma cancers (NSCLC). Nine of 11 lung squamous cell carcinoma (LSCC) samples, and 11 of 14 lung adenocarcinoma samples used for IHC analysis (Table 1

**Table 1:** IHC analysis of L523S

**Tissues**	**Staining**	**Comments**
Squamous tumour	Positive	11 out of 12
Adenocarcinoma	Positive	12 out of 14
Adrenal	Negative	
Artery – endothelium	Negative	
Urinary Bladder	Negative	
Blood (bone marrow)	Negative	
Brain (basal ganglia)	Negative	
Brain (cortex)	Negative	
Breast	Negative	
Colon	Positive (1 out of two)	Mild cytoplasmic staining of colonic epithelial cells
Oesophagus	Negative	
Fallopian tube	Positive	Mild cytoplasmic staining of epithelium
Gall bladder	Positive	Mild cytoplasmic staining of epithelium
Heart	Negative	
Kidney	Negative	
Liver	Negative	
Lung	Negative (zero out of six)	
Lumph node	Negative	
Ovary	Positive	Follicular epithelium marginally label; otherwise negative
Pancreas	Negative	
Parathyroid	Negative	
Pituitary	Positive	Mild cytoplasmic staining of cells in the pars anterior
Parathyroid	Negative	
Pituitary	Positive	Mild cytoplasmic staining of cells in the pars anterior
Placenta	Positive	Syncytial trophoblasts of terminal villi
Prostate	Negative	
Skeletal muscle	Negative	
Skin	Negative	
Small bowel	Negative	
Spinal cord	Negative	
Spleen	Negative	
Stomach	Negative	
Testes	Negative	
Thymus	Negative	
Thyroid	Negative	
Ureter	Negative	
Uterus	Negative	
Bronchus	Positive	Mild staining of columnar, ciliated epithelium
Trachea	Negative	Epithelium not present; otherwise negative
Tonsil	Positive	Staining of some germinal control cells/epithelial crypts

) are from National Kinki-Chuo Hospital, Osaka, Japan. Among these samples, 12 are stage I tumours, four are stage II tumours, and four are stage III tumours.

### cDNA microarray analysis of L523S

**Figure 1:**
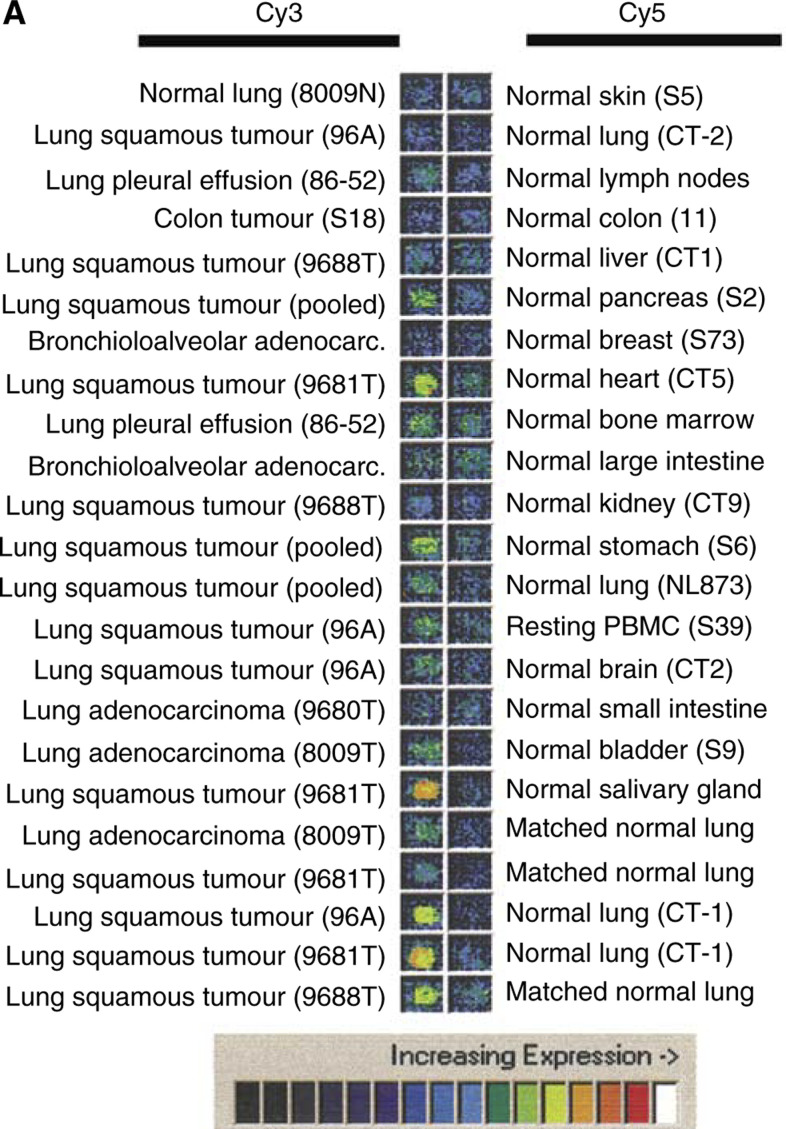

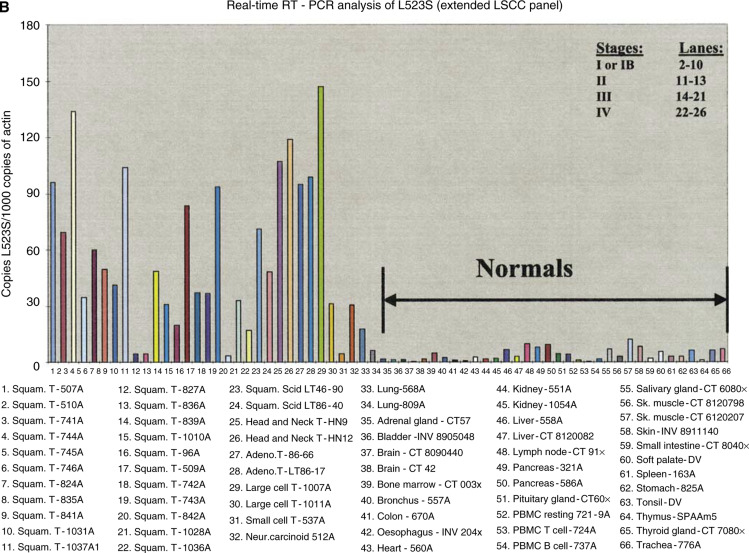
(**A**) Microarray analysis of L523S. Pseudocolour images of hybridisation intensities (white being the strongest and black being the weakest) were shown between 23 pairs of probes in Cy3 and Cy5 channels as indicated. (**B**) Real-time PCR analysis of L523S in NSCLC using an extended panel of 66 cDNA samples for LSCC. Overexpression is defined as ⩾10-fold expression compared with average expression in all normal tissues, or ⩾three-fold expression compared with highest expression in normal tissues. Overexpression of L523S was detected in 21 of 26 squamous tumours with 24 being LSCC. Expression of L523S was not associated with stages of the disease. (Figure continued on next page.)

As described previously in detail (Wang *et al*, 2000), L523S was identified as a gene overexpressed in LSCC using 23 pairs of cDNA probes synthesised from mRNA of lung tumour, normal lung, and other tumour and normal tissues. The expression profile for L523S was illustrated in pseudocolours representing pairs of Cy3 and Cy5 hybridisation signals, white being the highest and black being the lowest (Figure 1A

).

### Quantitative real-time PCR analysis

To compare the relative level of gene expression in multiple tissue samples, a panel of 36–66 cDNA samples was constructed using total RNA extracted from tissues and/or cell line(s), and real-time PCR was performed using gene-specific primers to quantify the copy number in each cDNA sample. Each cDNA sample was tested in duplicate and the final real-time PCR result was reported as an average of copy number of a gene of interest normalised against copy number of internal actin gene in each cDNA sample. Figure 1B shows as extended LSCC panel with various stages of tumours. Details of cDNA sources are also denoted. Specific primers used for real-time analysis are L523S-FW (5′ CATGGACTGGCTTTCTGGTTG 3′) and L523S-RV (5′CTGAGAAAAGCTCTGGCCTTAAAC3′). All real-time PCR reactions were run on a GeneAmp 5700 Detector using SYBR Green I dye (Perkin Elmer/Applied Biosystems Division, Foster City, CA, USA).

### Conventional RT–PCR analysis

Normalised mouse cDNAs from heart, brain, spleen, lung, liver, skeletal muscle, kidney, testis, 7, 11, 15, and 17-day embryo tissues were purchased through Clontech (Palo Alto, CA, USA). Each human cDNA used is also normalised by internal actin copy numbers. L523S universal primers (forward: 5′ ATGAATCTTCAAGCACATTTA 3′, and reverse: 5′ TCTCATCAGGTGTCTGGTCAC 3′) are 100% conserved between human L523S cDNA and its mouse homologue. Standard PCR analysis was performed (35 cycles).

### L523S protein expression and polyclonal antibody generation

*Escherchia coli* recombinant L523S protein was expressed in pPDM His, a modified pET28 vector (Novagen Inc., Madison WI, USA). Amino acid 2–580 of L523S was expressed in frame with an N-terminal His tag (MQHHHHHH). L523S protein was purified through nickel-affinity chromatography and ion-exchange chromatography according to standard protocols. The purified L523S protein was subjected to N-terminal sequencing analysis to confirm its identity. Rabbit anti-L523S polyclonal antisera were generated and affinity purified. Anti-L523S F(ab) fragment of IgG molecule was generated by cleavage with 0.5 mg ml^−1^ papain at 37°C for 2 h followed by protein A removal of uncleaved and cleaved Fc region and dialysis in PBS.

### Western blot and immunohistochemistry analysis

Total cellular lysate was prepared by homogenising small pieces of snap-frozen tissues in three-volume lysis buffer (250 mM sucrose, 20 mM HEPES, pH7.5, and 1 mM EDTA), followed by 5 min centrifugation with 5000 r.p.m. at 4°C. 10 *μ*g of total protein lysate was loaded in each lane on two SDS–PAGE gels with 10 ng recombinant L523S used for positive control. After electrophoresis, one gel was stained with Commassie blue, and the other was electroblotted onto a nitrocellular membrane followed by primary antibody (1 : 10 000 dilution of anti-L523S polyclonal antibody) and secondary antibody (1 : 5000 dilution of HRP-conjugated goat anti-rabbit) incubation. The blot was developed by ECL reagents. Immunohistochemsitry analysis was performed with affinity-purified anti-L523S polyclonal antibody. In all cases, 4 *μ*m sections of formalin-fixed, paraffin-embedded tissues were used. Tissues were subjected to 20 min steam-heat-induced epitope retrieval in presence of 10 mM sodium citrate buffer (pH 5.6–6.0) before being stained with either 1 *μ*g ml^−1^ of IgG or 4.0 *μ*g ml^−1^ F(ab) anti-L523S rabbit polyclonal antibody. Tissues were then incubated with a biotinylated anti-rabbit secondary antibody. After endogenous peroxidase blocking, the avidin–biotin complex/HRP (ABC/HRP) was used along with DAB chromogen to visualise protein expression.

### ELISA and Western blot analysis using lung pleural effusion from lung cancer patients

ELISA was performed using recombinant L523S protein with lung pleural effusion fluids from lung cancer patients and sera from normal donors following a standard ELISA protocol. Briefly, a 96-well microtitre plate was coated with 350 ng well^−1^ L523S protein overnight at 4°C and blocked by 10% nonfat dry milk in PBS for 3 h at room temperature. Pleural fluid titres from 17 NSCLC patients as well as 48 sera from normal donors were tested at 1 : 30, 1 : 100, 1 : 300, and 1 : 1000 dilutions, in PBS with 5% goat sera and 5% nonfat dry milk for 3 h at room temperature. After 30 min secondary antibody incubation with 1 : 8000 of HRP-conjugated donkey anti-human IgG+IgM (Jackson ImmunoResearch Lab., West Grove, PA, USA), the plate was developed by ortho-phenylendiamine and hydroperoxide. The Western blot analysis was performed as described above using 1 *μ*g recombinant L523S and 1 : 30 to 1 : 300 dilutions of patient pleural effusions.

## RESULTS

### L523S expression in lung cancer

Microarray analysis of L523S using 23 pairs of cDNA probes synthesised from polyA+RNA of tumour and normal tissues showed elevated L523S expression in four of four LSCC samples (pooled, 9681T, 9688T, and 96A) with little or no expression detected in normal tissues (Figure 1A). Two of three lung adenocarcinoma cDNA samples also showed increased expression of L523S, but at a lower level (pleural effusion 86–52 and 8009T). The expression profile of L523S observed through microarray analysis was confirmed by real-time PCR analysis using cDNA panels comprised of samples from both LSCC and lung adenocarcinoma (data not shown). In order to determine the frequency of L523S expression in LSCC and adenocarcinoma, as well as to assess potential correlation of L523S expression with stages of each histological type of lung cancer, we examined the L523S expression in an extended cDNA panel using real-time PCR. The LSCC panel contained 26 cDNA samples derived from stage I to stage IV tumours, including two head and neck samples, and the lung adenocarcinoma panel comprised 25 cDNAs derived from adenocarcinoma of different stages. As shown in Figure 1B, L523S is overexpressed in 21 of 26 squamous cell carcinoma samples and at all tumour stages. L523S expression in lung adenocarcinoma appeared to be more heterogeneous, again with no correlation with disease stage, but in a lower overall proportion of tumours (seven of 26 lung adenocarcinoma samples, data not shown). Expression of L523S in 34 normal tissues was found to be minimal when examined on normal tissue cDNA panels by real-time PCR (Figure 1B).

### L523S is a highly conserved oncofetal protein

**Figure 2:**
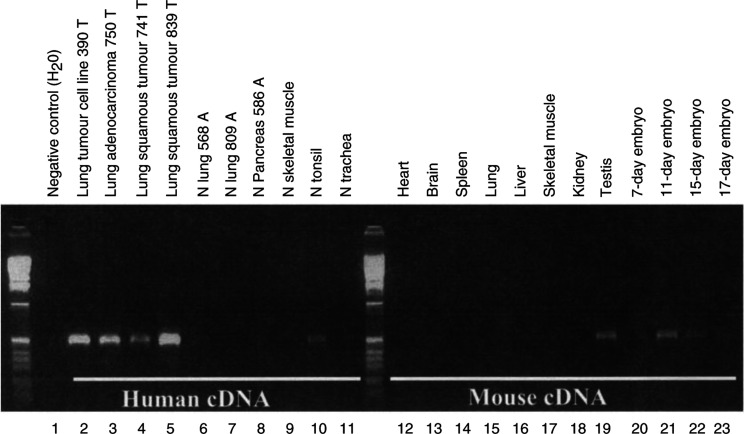
L523S is overexpressed in tumour and embryonic tissues. RT–PCR analysis of L523S expression in human tissues (lanes 2–11) and its homologue in mouse tissues (lanes 12–23) was carried out under the same condition in a single experiment. Human cDNA was normalised by internal actin at cycle numbers 20–22, and mouse cDNA was prenormalised by the manufacturer (Clontech, Palo Alto, CA, USA). Primers were designed to have 100% identity to both human and mouse L523S cDNA sequences (see Materials and Methods for detail).

Oncofetal protein are by definition proteins that are normally expressed only during embryonic development and then re-expressed in cancers. Some oncofetal protein are also expressed in placenta and testis tissues. Earlier studies have demonstrated that an L523S mouse homologue (96% identical at the protein level) is ubiquitously expressed during early stages of mouse embryogenesis (Mueller-Pillasch *et al*, 1999), indicating an important role for L523S protein during early stages of embryonic development. Since human and mouse L523S homologues are also highly conserved at the cDNA level (92% identical), we investigated whether L523S expression in lung cancer is at a level similar to that of expression during embryogenesis. Using primers that recognise both human and mouse L523S transcripts, we show by PCR analysis that L523S, consistent with the definition of an oncofetal antigen, is expressed in lung cancers (Figure 2

, lanes 2–5) and early mouse embryos and mouse adult testis (Figure 2, lanes 19–23). In contrast, we found little or no expression detected in normal adult tissues of human (Figure 2, lanes 6–11) or mouse (Figure 2, lanes 12–18).

### L523S protein is localised in the cytoplasm of lung cancer cells

**Figure 3:**
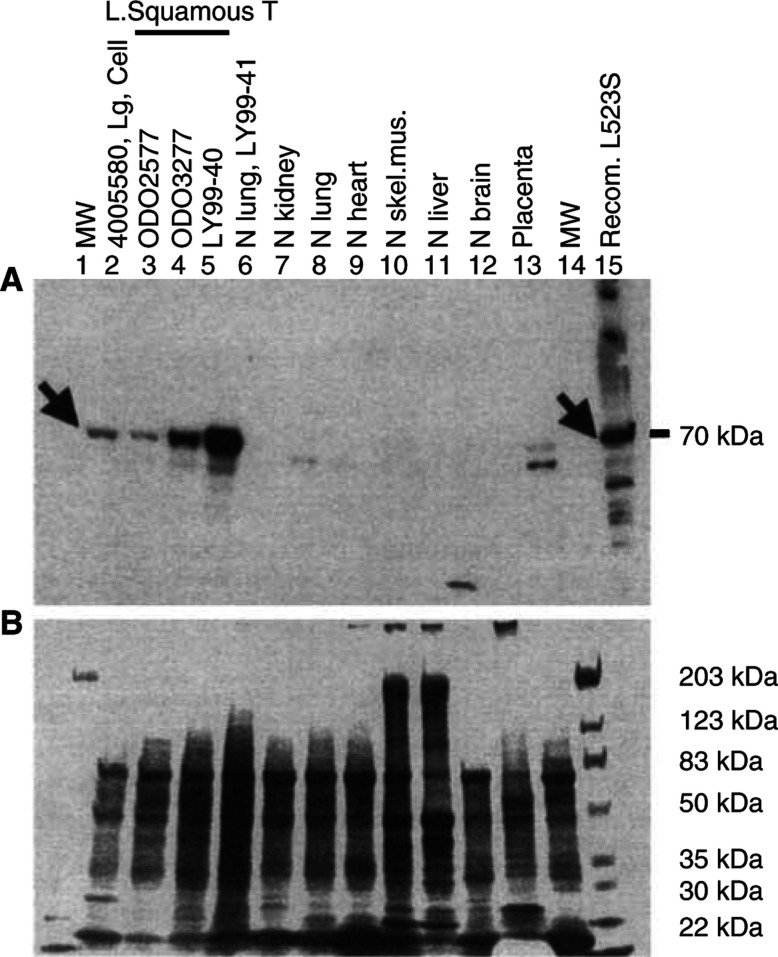
Western blot analysis revealed a 70 kDa L523S protein in one large cell carcinoma and three LSCC tissues (**A**). This 70 kDa protein was not detected in any other normal tissues except for a weaker signal in the placenta tissue. Notice that there was a lower molecular weight protein present in normal kidney and placenta tissues, and a 20 kDa band was also visible in the liver sample. Parallel gel with total proteins from cell lysate was stained with Commassie (**B**).

Western blot analysis using affinity-purified L523S polyclonal antibodies revealed that the L523S protein was present in lung cancer samples. By using whole cell lysate extracted from tissues of lung cancer and normal adult tissues, L523S protein was detected in a large cell carcinoma and three LSCC samples (Figure 3A

, lanes 2–5). A weaker L523S signal was also detected in a placenta tissue sample (Figure 3A, lane 13).

**Figure 4:**
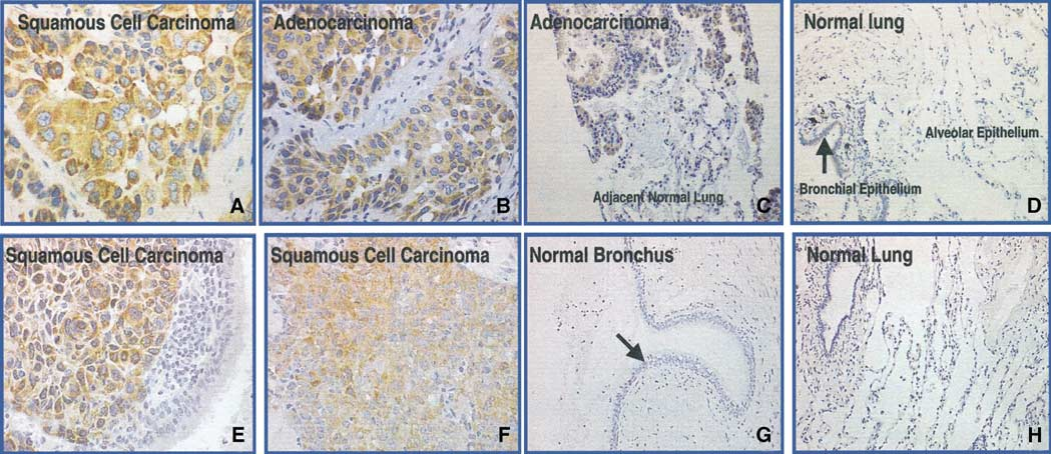
Immunohistochemistry analysis of L523S using affinity-purified whole IgG molecules (**A**–**D**) and F(ab) fragments (**E**–**H**). Illustrated here are results of IHC staining on tissue sections of LSCC (**A**, **E**, **F**), lung adenocarcinoma (**B**), lung adenocarcinoma with adjacent normal lung tissue (**C**), and normal tissues of lung (**D**, **H**). Homogeneous cytoplasm staining was observed in both squamous and adenocarcinoma samples (**A**–**C**, **E**, **F**). Arrows point to bronchiole epithelial cells that were lightly stained with whole IgG molecules (**D**), but not with F(ab) fragments (**G**).

Immunohistochemistry (IHC) analysis demonstrated that L523S is present homogeneously in the cytoplasm of lung cancer cells, both in squamous cell carcinoma (Figure 4A

) and in adenocarcinoma (Figure 4B). As shown in Figure 4C, L523S protein was only detected in the tumour portion of the adenocarcinoma tissue section, not in adjacent normal lung tissue, suggesting that its expression is specifically associated with malignant cells. The results of IHC analysis using an extensive panel of lung cancer and normal tissues are summarised in Table 1. In total, 90% of LSCC and adenocarcinoma scored positive for L523S immunoreactivity. Although real-time PCR analysis showed that L523S mRNA levels vary in lung adenocarcinoma samples (data not shown), the IHC analysis clearly demonstrated that L523S protein is present in a high percentage of lung adenocarcinoma. Consistent with mRNA expression profiles, L523S immunoreactivity was not found in the majority of normal adult tissues; however, light staining was detected in a few normal epithelial cell types including fallopian tube, gall bladder and normal bronchiolar epithelial cells (Table 1 and Figure 4D). Since the faint immunoreactivity observed in normal epithelial cells might have resulted from nonspecific binding of the Fc region of the IgG molecule, we performed IHC analysis using purified F(ab) fragment of the IgG molecules (Figure 4E–H). Under similar staining conditions, immunoreactivity of L523S in normal bronchiolar epithelial cells was greatly diminished (Figure 4G) with no apparent loss in signals for L523S in two LSCC (Figure 4E, F), although the background under previous condition is slightly higher. Nonetheless, L523S protein is overexpressed in lung cancer and appears to be localised to the cytoplasm of cancerous cells.

### Antibody responses to L523S in lung cancer patients

To test whether L523S protein is immunogenic in lung cancer patients, an ELISA was performed using recombinant L523S protein to test for the presence of antibody responses in lung pleural effusions from 17 patients (Table 2

**Table 2:** Antibody responses to L523S in lung cancer patients

**Lung pleural effusion from patients**	**Responses to BSA**	**Responses to L523S**
1	−	−
2	−	−
3	−	−
4	−	++
5	−	++
6	−	−
7	−	+++
8	−	++
9	−	++
10	−	+++
11	−	−
12	−	+++
13	−	−
14	−	−
15	−	++
16	−	−
17	−	−
		
48 sera from nomal donors		−

**Figure 5:**
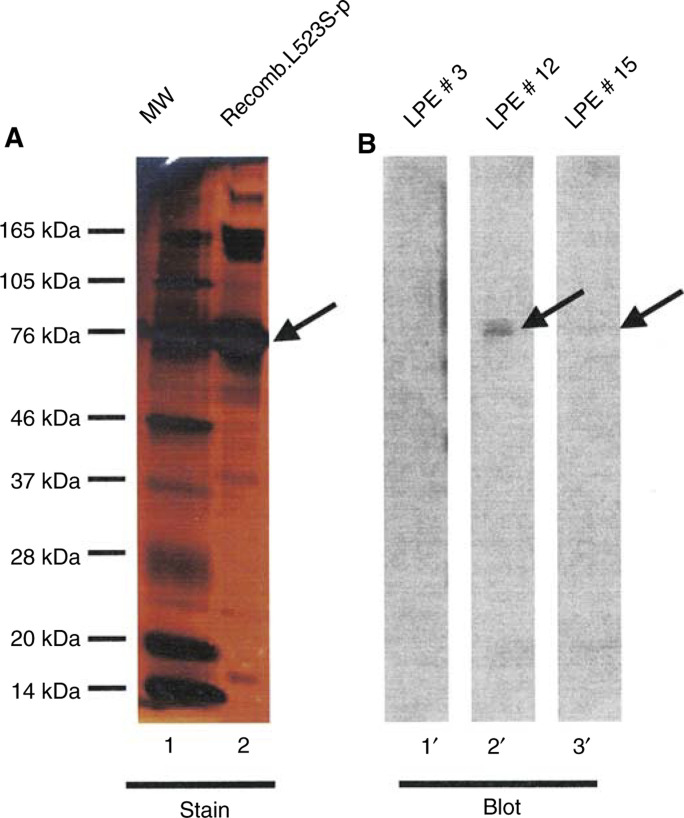
Western blot analysis of immunoreactivity of lung cancer patient antibody to L523S. (**A**) Silver staining of recombinant L523S protein purified through nickel chromatography (lane 2). The arrow in (**A**) points to L523S protein confirmed by N-terminal sequencing analysis. (**B**) L523S recombinant protein was probed with lung pleural effusion from patient #3 (lane 1′), #12 (lane 2′), and #15 (lane 3′). Notice that stronger antibody titre in #12 detected by ELISA analysis (Table 2) also has increased immunoreactivity to L523S protein by Western blot analysis.

The data are derived from ELISA. The specificity of the antibody response in each ELISA-positive sample was also confirmed by Western blot analysis. ‘++’ Indicates that Western blot result was obtained with more than 30-fold dilution of LPE, whereas ‘+++’ denotes immunoreactivity by Western blot with more than 300-fold dilution of the LPE samples.

) (Rohrer *et al*, 1999; Vollmer *et al*, 1999). Eight pleural effusions from lung cancer patients gave positive signals (>two-fold signals in at least two different titres compared with normal sera of 48 donors). These positive signals are significant, as the immunoreactivity could not be abolished by using *E. coli* protein lysates as competitors. Strong antibody titres were observed in eight patients with no positive signals detected in the sera of 48 normal donors (Table 2). Although antibody titre in individual lung pleural effusions was variable, the specificity of antibody response in each patient was confirmed by Western blot analysis. Representative Western blot analysis of lung pleural effusion antibody responses in two positive and one negative patient is shown in Figure 5

. L523S-specific antibody reactivity was present in patient pleural effusion #12 and #15 but not #3 (Table 2 and Figure 5). Other studies performed by us (R Houghton and DH Persing, and others) have shown that sera from patients with lung and other malignancies show reactivity to L523S (unpublished observations).

## DISCUSSION

In this report, we show that L523S is an oncofetal protein and is overexpressed in lung squamous and adenocarcinoma tumours. L523S is expressed in 80–90% of LSCC by mRNA measurements as well as by IHC analysis. In addition, L523S is also expressed in a significant majority of lung adenocarcinoma, making it an attractive therapeutic target with broad coverage in NSCLC. Similar to other ‘cancer antigens’ such as NY-ESO-1 (Jager *et al*, 1998), immunological tolerance to L523S at the humoral level appears to be naturally overcome in a significant proportion of lung cancer patients, suggesting a role for antigen-presenting cells, helper cells, and B cells in facilitating such responses. Since expression of L523S appears to be extremely limited in adult normal tissues, humoral and/or cellular immune responses directed against L523S are likely to be tumour specific and perhaps less likely to trigger autoimmune responses. Indeed, limited normal tissue expression of L523S may account for the high rate of antibody positivity found in cancer patients (eight out of 17 pleural effusion samples from cancer patients), given the limited immunological tolerance exhibited toward oncofetal antigens in general. At the very least, our studies suggest that immune responses against L523S, at the humoral level, are not incompatible with life. We did not address the possibility that some of the negative samples in this analysis actually comprised immune complexes with L523S antigen, thus rendering the antibody response less detectable, but this is a distinct possibility given the high rate of L523S antigen positivity in tumour samples, coupled with the likely presence of cellular antigens from necrotic tumours within the pleural exudates.

Some studies have shown that humoral and cellular responses to tumour-associated antigen are correlated with each other (Jager *et al*, 1998). However, we are also well aware that patients who had humoral responses to self-antigens such as L523S went on to have progressive diseases, suggesting that perhaps an enhancement of cellular immunity, as mediated by cytotoxic T-cell responses, is more important to resolve the tumour burden. Studies of L523S immunogenicity using *in vitro* human T-cell priming are currently underway, and *in vivo* immunogenicity studies are also being carried out to measure specific cellular and humoral responses against L523S by vaccination of mice. We are fortunate that the mouse homologue of L523S is highly conserved relative to the cognate human sequence, so that immunisation studies in the mouse are likely to be relevant to the issue of vaccine safety. It is interesting to note that L523S prevalence is not restricted to lung cancer, and that L523S was also found to be overexpressed in pancreatic cancers (Mueller-Pillasch *et al*, 1997). Thus, the therapeutic range of L523S may extend to other cancer types.

It is intriguing that L523S, as an oncofetal RNA-binding protein, is re-expressed in cancerous cells. RNA-binding proteins play important roles in the post-transcriptional regulation of gene expression and are known for controlling the localisation, stability, and translation of mRNA, particularly during early stages of embryogenesis. Post-transcriptional regulation of gene expression offers an advantage for genes whose expression is controlled in according to a temporal or spatial program (Siomi and Dreyfuss, 1997). Although many studies on RNA-binding proteins have focused on genes involved in development and differentiation (Oostra, 2002; Reijo *et al*, 1995), less is known about their roles in tumourigenesis. Recent studies revealed that multiple embryonic RNA-binding proteins are overexpressed in several different cancer types (Mueller-Pillasch *et al*, 1997; Wang *et al*, 2000). It is possible that expression of L523S is necessary for maintenance of the transformed state, which can potentially be determined by gene knockout studies such as those performed by RNA interference (RNAi). If it is indeed shown to be essential, its value as an antitumour target could be enhanced, since immunological escape variants might be less likely to develop. In addition, it could become a preferred target for small molecule development.

p62 Protein, a homologue of L523S with 80% overall sequence similarity, was found to be an immunogenic autoantigen in human liver cancers (Zhang *et al*, 1999). In contrast to L523S, the p62 gene is expressed in a variety of normal tissues including kidney, stomach, pancreas, and liver when its mRNA expression is measured against LSCC (data not shown). However, it is likely that p62 expression in liver cancers is much higher and more prevalent. The lower molecular weight protein detected in kidney and placenta could be to L523S antibody crossreactivity with the p62 protein (Figure 3), although recent data have shown autoantibodies from patients have minimal crossreactivity between L523S and p62 (Zhang *et al*, 2001). L523S and p62 were also discovered independently as insulin-like growth factor II mRNA-binding proteins, known as IMP-3 and IMP-2, respectively (Nielsen *et al*, 1999). It will be important to determine if overexpression of L523S or p62 in cancer cells plays any role in regulation of transcripts from insulin-like growth factor II or other growth-related genes. In conclusion, our studies indicate that L523S may be a valuable addition to the repertoire of cancer-specific targets for the development of new immunotherapeutic and perhaps other therapeutic approaches.
